# LDL cholesterol burden in elderly patients with familial hypercholesterolemia: Insights from real-world data

**DOI:** 10.1016/j.ajpc.2025.100986

**Published:** 2025-03-29

**Authors:** Torunn Melnes, Martin P. Bogsrud, Jacob J. Christensen, Amanda Rundblad, Kjetil Retterstøl, Ingunn Narverud, Pål Aukrust, Bente Halvorsen, Stine M. Ulven, Kirsten B. Holven

**Affiliations:** a Department of Nutrition, Institute of Basic Medical Sciences, University of Oslo, Oslo, Norway; b Unit for Cardiac and Cardiovascular Genetics, Department of Medical Genetics, Oslo University Hospital Ullevål, Norway; c Norwegian National Advisory Unit on Familial Hypercholesterolemia, Department of Endocrinology, Morbid Obesity and Preventive Medicine, Oslo University Hospital Aker, Norway; d The Lipid Clinic, Department of Endocrinology, Morbid Obesity and Preventive Medicine, Oslo University Hospital Aker, Norway; e Research Institute for Internal Medicine, Oslo University Hospital, Norway; f Institute of Clinical Medicine, University of Oslo, Norway

**Keywords:** Familial hypercholesterolemia, Elderly, Cholesterol burden, Cumulative LDL-C exposure, Coronary heart disease

## Abstract

**Background and aims:**

Familial hypercholesterolemia (FH) is a genetic disorder characterized by elevated low-density lipoprotein cholesterol (LDL-C) and increased risk of premature coronary heart disease (CHD). While current LDL-C levels usually guides therapy, the cumulative exposure to LDL-C (the LDL-C burden) is suggested to offer a more precise estimate of cardiovascular risk in people with FH. Therefore, using real-world data, this study aimed to estimate the LDL-C burden at different ages in elderly FH patients with and without CHD, and to assess the LDL-C burden at CHD onset.

**Methods:**

Data was retrospectively collected from the medical records of elderly (>60 years) FH patients at the Lipid Clinic in Oslo. The LDL-C burden (mM-years) was estimated based on repeated LDL-C measurements and information on lipid-lowering medication. Time-weighted average (TWA) LDL-C was calculated as LDL-C burden divided by years.

**Results:**

We included 112 FH patients, of which 55 (49 %) had experienced at least one CHD-event, and 58 (52 %) were females. Median age at first and last visit were 50 years and 68 years, respectively, with a median of 9 (range; 2–14) available LDL-C measurements. Subjects with CHD had higher LDL-C burden at all ages tested (45, 50 and 60 years) compared with the non-CHD group (*p* < 0.01, also after adjusting for sex), and had higher TWA LDL-C before treatment at the Lipid Clinic (*p* = 0.004), but not during follow-up (*p* = 0.6). There were no sex differences in LDL-C burden at all ages tested, also after adjusting for CHD (*p* > 0.1). However, women had higher TWA LDL-C during follow-up at the Lipid Clinic (*p* = 0.01). Median LDL-C burden at CHD onset was 352 mM-years; numerically lower in women than in men (320 vs. 357 mM-years, respectively. *p* = 0.1).

**Conclusion:**

Elderly FH patients with CHD had higher estimated LDL-C burden compared with FH patients without CHD, due to higher burden *prior to treatment*, highlighting the importance of early

detection and treatment.

## Graphical abstract

**Figure:**
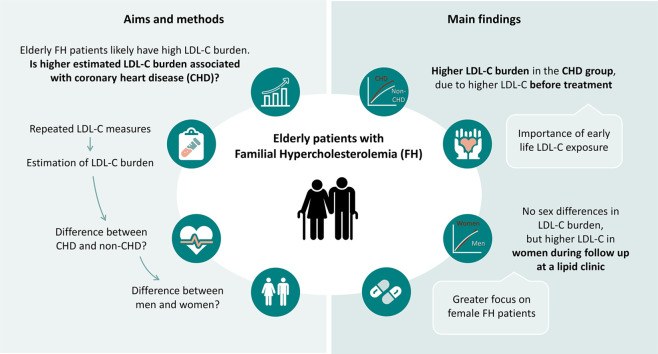

## 1 Introduction

Low-density lipoprotein cholesterol (LDL-C) is one of the main drivers of atherosclerosis and a causal risk factor for atherosclerotic cardiovascular disease (ASCVD) [1,2], thus lowering of LDL-C remains the main focus in ASCVD prevention [3,4]. However, not only the current LDL-C levels are of importance. Because the atherosclerotic plaque develops over time, the duration and magnitude of the total LDL-C exposure to the arterial wall defines the risk of ASCVD [[5], [6], [7], [8]]. Recent studies suggest that the total LDL-C exposure, or LDL-C burden, is associated with coronary heart disease (CHD) independent of current LDL-C levels [[9], [10], [11]].

Patients with familial hypercholesterolemia (FH) have elevated LDL-C from the first year of life, leading to an increased risk of premature CHD. The increased LDL-C concentration is caused by mutation in genes involved in cholesterol metabolism, mostly *LDLR, APOB* or *PCSK9*, affecting liver LDL-C uptake from the circulation [12]. Lipid-lowering therapies such as statins, ezetimibe and PCSK9 inhibitors, reduce the LDL-C and, hence, the risk of CHD in FH patients [12]. However, individuals carrying an FH mutation have increased cardiovascular risk compared to those without mutation with similar current LDL-C levels, due to a higher accumulated cholesterol burden earlier in life, emphasising the importance of high lifetime LDL-C burden in FH patients [11]. Indeed, recent studies have shown that estimation of LDL-C burden provides an even better risk prediction in FH patients than single LDL-C levels [13], and that every 75 mM-years increase in LDL-C burden is associated with a doubling in total plaque volume [14]. While theoretical thresholds for LDL-C burden at which the risk of myocardial infarction (MI) starts to increase (125 mM-years or 5000 mg/dL-years) and an average LDL-C burden at which MI occurs (210 mM-years or 8000 mg/dL-years) have been suggested for the general population [5], it is unknown if these threshold values can be relevant for FH patients.

FH patients diagnosed before effective cholesterol-lowering medication was available, or diagnosed late in life, thus untreated for several years, represent a group of patients with *extremely* high LDL-C burden and a very high risk of CHD. Still, some of these patients do not experience CHD. An estimation of LDL-C burden in such FH patients remains to be calculated, both in individuals with CHD and in those who surprisingly remain free from CHD. In this study, we used a group of elderly FH patients with several LDL-C measurements throughout many years of follow-up at the Lipid Clinic in Oslo, to estimate “real-life” LDL-C burden. The aim was to compare LDL-C burden in elderly FH patients with and without CHD, compare LDL-C burden in women and men, and to assess the LDL-C burden at which the first CHD-event occurred.

## 2 Methods

### 2.1 Participants

We retrospectively collected data from the medical records of elderly FH patients at the Lipid Clinic, Oslo University Hospital (OUH), Norway. FH patients were included if they were born in 1952 or earlier, had a definite FH diagnosis, and had at least two visits with available LDL-C data with the first measurement at the age of 60 years or younger. Participants had their last visit at the Lipid Clinic during 2014–2017, and data was collected until December 2017. FH diagnosis was based on genetic testing or a Dutch Lipid Clinic Network score above 8. Presence of CHD was defined as MI, angina pectoris, percutaneous coronary intervention, and/or coronary artery bypass surgery. Potent statin was defined as use of at least 20 mg Rosuvastatin, 40 mg Atorvastatin or 80 mg Simvastatin. The study was approved by the regional ethical commiteee.

Most FH patients (79 [71 %] of total 112) had available LDL-C concentration from the first visit at the Lipid Clinic. If LDL-C was missing at first visit at the Lipid Clinic, but total cholesterol (TC), high-density lipoprotein cholesterol (HDL-C) and triglycerides (TG) (<4.5 mmol/L) were available (in 21 patients [19 %]), we calculated LDL-C using Friedewald's equation: TC – HDL – TG/2.2 [15]. For the remaining 12 patients (10 %) without LDL-C or possibility to use Friedewald's equation, the first available LDL-C measurement was from the second visit at the Lipid Clinic or later.

### 2.2 Estimation of LDL-C burden

To estimate the LDL-C burden (mM-years), we used repeated LDL-C measurements and information regarding lipid-lowering medication for each participant. Mainly, each LDL-C concentration (mM) was multiplied by the number of years since the previous measurement [16], as illustrated in **Supplementary Figure 1**. The very first available LDL-C was multiplied by the age at that time point.

### 2.3 Estimation of untreated LDL-C

For participants already using statins at the time of first LDL-C measurement, we calculated an untreated LDL-C, and multiplied it with the age at statin start. Due to lack of detailed information on statin type and dose at first LDL-C measure, the calculation of untreated LDL-C was performed in two different ways, to provide the best estimate. First, the first measures of LDL-C for statin treated patients were adjusted to an LDL-C reduction of 30 % as previously described by Langsted et al. [17,18]. Second, we used the validated algorithm by Ruel et al. [19] to calculate untreated LDL-C based on type and dose of statin and Ezetimibe at the *last* LDL-C measurement. The results generated were quite similar and highly correlated, except a slightly higher estimation of LDL-C burden for all groups using the Ruel algorithm, thus the first approach (30 % LDL-C reduction for all that needed calculation of untreated LDL-C) was used for the analyses.

### 2.4 Time-weighted average LDL-C

To be able to compare LDL-C burden at CHD onset, before treatment, and during follow-up at the Lipid Clinic despite varying ages, we calculated the time-weighted average (TWA) LDL-C [9]. This was done by dividing estimated LDL-C burden by age at same time point. As the unit of TWA LDL-C is mmol/L which is used for single LDL-C measures, this approach is likely more understandable for clinicians and patients, and therefore potentially useful in clinical settings.

### 2.5 Statistical analysis

Characteristics of the participants were presented as median and 25–75 percentiles. To compare groups (CHD vs. non-CHD, women vs. men, and lowest vs. highest LDL-C burden at CHD onset), we used Mann-Whitney *U* test for continuous variables and Chi-square test for categorical variables.

For comparison of LDL-C burden and TWA LDL-C between the groups at certain ages (45 years, 50 years, and 60 years), at onset of CHD, and for TWA LDL-C before and during follow-up at the Lipid Clinic, we used Mann-Whitney *U* test. To adjust for covariates in the same comparisons, we performed linear regression models with LDL-C burden and TWA LDL-C as outcomes, groups as exposure, and sex, CHD, or age at CHD onset as covariates.

To compare the two different approaches to calculate untreated LDL-C, we calculated Pearson correlation coefficient.

All statistical analyses were performed in R (version 4.3.1) using RStudio IDE (version 2023.06.0 + 421, posit.co) [20]. Tests were two-sided, and significance level was set to 5 %.

## 3 Results

### 3.1 Characteristics of the study population

We included 112 FH patients, of which 55 (49 %) had experienced at least one CHD-event, and 58 (52 %) were females. Median age was 50 years (25–75 percentile; 43–55) at first visit and 68 years (66–71) at last visit. While there was no difference in the duration of follow-up at the Lipid Clinic for FH patients with and without CHD (see Table 1), the CHD group had more visits and thus more LDL-C measurements (11 vs. 9 visits, *p* = 0.003; and 10 vs. 9 LDL-C measurements, *p* = 0.039). There were fewer females in the CHD group compared with the non-CHD group (38 % vs. 65 %, *p* = 0.005).

**Table 1:** Characteristics of elderly FH patients with and without CHD.

	**n**	**Overall***n* = 112	**CHD***n* = 55	**Non-CHD***n* = 57	***p***
**Characteristics**					
Female, n (%)	112	58 (52)	21 (38)	37 (65)	**0.005**
Age last visit, years	112	68 (66–71)	69 (66–72)	68 (65–71)	0.3
Age first CHD-event	55	50 (43–56)	50 (43–56)	–	–
CHD before Lipid Clinic, n (%)	55	29 (53)	29 (53)	–	–
Premature CHD^a^, n (%)	112	41 (37)	41 (75)	0	**<0.001**
Genetically confirmed FH, n (%)	112	109 (97)	54 (98)	55 (96)	>0.9
BMI^b^, kg/m^2^	95	26.5 (24.0–29.1)	27.1 (25.5–31.5)	26.0 (23.8–28.4)	0.12
**Medication use**					
Statin use first visit, n (%)	106	55 (52)	28 (54)	27 (50)	0.7
Statin use last visit, n (%)	110	101 (92)	48 (89)	53 (95)	0.3
Potent statin^b^^,^^c^, n (%)	110	84 (76)	40 (74)	44 (79)	0.6
Age at statin start, years	88	45 (40–50)	44 (40–50)	45.0 (41–51)	0.5
**Follow-up at the Lipid Clinic**					
Age first visit, years	112	50 (43–55)	49 (42–55)	50 (43–55)	0.4
Age first LDL-C measure, years	112	50 (44–55)	49 (44–55)	51 (44–55)	0.5
Follow-up, years	112	21 (15–27)	22 (17–28)	18 (14–27)	0.11
Number of visits, n	112	10 (7–14)	11 (8–16)	9 (6–12)	**0.003**
Number of LDL-C measurements, n	112	9 (8–11)	10 (8–11)	9 (7–10)	**0.039**

Data are presented as n (%) for categorical variables and median (25th-75th percentiles) for continuous variables. Differences between CHD and non-CHD are tested with chi-square test for categorical variables, and Mann-Whitney *U* test for continuous variables. Abbreviations: CHD, coronary heart disease; FH, familial hypercholesterolemia; BMI, body mass index.

a CHD <55 years in males; <65 years in females.

b Last visit at the Lipid Clinic.

c At least 20 mg Rosuvastatin, 40 mg Atorvastatin or 80 mg Simvastatin.

Regarding statin use, there were no differences between those with and without CHD in the proportion who used statins at first and last visit, no difference in use of potent statin at last visit, and no difference in age at statin start (see Table 1). However, more subjects in the CHD group used PCSK9 inhibitors at last visit (38 % vs. 14 %, *p* = 0.004, see **Supplementary Table 1**). Due to statin use at the time of first LDL-C measurement, we estimated the untreated LDL-C in 53 (47 %) FH patients (53 % and 42 % in patients with and without CHD, respectively [*p* = 0.3]) to further calculate the LDL-C burden.

### 3.2 Cardiometabolic markers in the CHD and non-CHD groups

Details on lipid profile and cardiometabolic markers at first and last visit in the two FH groups are shown in Table 2. Briefly, the CHD group had a more atherogenic lipid profile at the first visit at the Lipid Clinic. At last visit, both groups had reduced TC and LDL-C, and TC was indeed lower in the CHD group compared to non-CHD (4.30 mmol/L vs. 4.85 mmol/L, *p* = 0.036). However, TG were still higher in the CHD group at last visit (1.10 mmol/L vs. 0.90 mmol/L, *p* = 0.038), and high-density lipoprotein cholesterol (HDL-C) remained lower (1.30 mmol/L vs. 1.50 mmol/L, *p* = 0.016). Only 8 (15 %) and 18 (33 %) patients with CHD reached a treatment target of ≤1.4 and ≤1.8 mmol/L, respectively. In contrast, among the patients without CHD, 26 (46 %) reached a treatment target ≤2.5 mmol/L.

**Table 2:** Biochemical measurements of elderly FH patients with and without CHD.

	**n**	**Overall** *n* = 112	**CHD** *n* = 55	**Non-CHD** *n* = 57	***p***
**First visit**					
TC, mmol/L	111	8.40 (6.45–10.10)	8.75 (6.80–11.48)	8.00 (6.10–9.50)	**0.029**
TC, highest measured, mmol/L	111	11.00 (9.45–12.70)	12.00 (10.63–13.00)	10.00 (9.00–11.50)	**<0.001**
LDL-C, mmol/L	100	6.20 (4.50–7.95)	6.60 (4.58–9.18)	5.85 (4.28–7.70)	**0.040**
LDL-C first measure^a^, mmol/L	112	6.00 (4.50–7.78)	6.20 (4.50–9.00)	5.70 (4.30–7.50)	0.13
HDL-C, mmol/L	104	1.30 (1.10–1.50)	1.20 (1.00–1.40)	1.40 (1.20–1.60)	**0.007**
TG, mmol/L	99	1.11 (0.77–1.50)	1.28 (0.90–1.89)	0.85 (0.70–1.23)	**<0.001**
**Last visit**					
TC, mmol/L	111	4.60 (3.75–5.25)	4.30 (3.60–5.05)	4.85 (4.15–5.43)	**0.036**
LDL-C, mmol/L	112	2.45 (1.80–3.30)	2.30 (1.75–3.15)	2.70 (2.10–3.30)	0.12
HDL-C, mmol/L	111	1.40 (1.20–1.70)	1.30 (1.10–1.65)	1.50 (1.30–1.73)	**0.016**
TG, mmol/L	111	1.10 (0.80–1.50)	1.10 (0.90–1.60)	0.90 (0.70–1.40)	**0.038**

Data are presented as median (25th-75th percentiles). Differences between CHD and non-CHD are tested with Mann-Whitney *U* test. Abbreviations: TC, total cholesterol; LDL-C, low-density lipoprotein cholesterol; HDL-C, high-density lipoprotein cholesterol; TG, triglycerides.

a First available LDL-C measurement.

### 3.3 Sex differences, CHD and lipid-lowering drugs

Table 3 and **Supplementary Table 2** shows sex differences in the CHD group only and in all FH patients, respectively. Briefly, fewer women than men had CHD (36 % vs. 63 %, *p* = 0.01), but among those with CHD, more women had premature CHD compared to men (90 % vs. 65 %, *p* = 0.033). While there were no difference in age at statin start between women and men (46 years vs. 45 years, *p* = 0.15), fewer female FH patients were treated with high-intensity statins than their male counterparts at last visit (66 % vs. 87 %, *p* = 0.01). This was also the case when we studied only the CHD group, where fewer women used Ezetimibe (65 % vs. 94 %, *p* = 0.009), resulting in higher on-treatment levels of TC (5.00 mmol/L vs. 3.80 mmol/L, *p* < 0.001) and LDL-C (2.90 mmol/L vs. 1.90 mmol/L, *p* < 0.001) at last visit at the Lipid Clinic.

**Table 3:** Characteristics of elderly female and male FH patients with CHD.

	**n**		**Women** *n* = 21		**Men** *n* = 34	***p***
**Characteristics**						
Age last visit, years	55		69.0 (67.0–72.0)		69.0 (66.0–71.8)	0.8
Age first CHD-event	55		45 (40–52)		52 (46–57)	0.13
Premature CHD^a^, n (%)	55		19 (90)		22 (65)	**0.033**
CHD before Lipid Clinic, n (%)	55		15 (71)		14 (41)	**0.029**
BMI^b^, kg/m^2^	47		25.6 (21.0–29.3)		27.6 (25.8–31.6)	0.055
**Medication use**						
Statin use first visit, n (%)	52		11 (58)		17 (52)	0.7
Statin use last visit, n (%)	54		16 (80)		32 (94)	0.2
Potent statin^b^^,^^c^, n (%)	54		11 (55)		29 (85)	**0.014**
Age at statin start, years	43		44.0 (41.0–50.0)		44.5 (40.0–49.3)	0.8
PCSK9 inhibitor^b^, n (%)	55		8 (38)		13 (38)	>0.9
Ezetimibe^b^, n (%)	54		13 (65)		32 (94)	**0.009**
Resin^b^, n (%)	53		3 (15)		10 (30)	0.3
**Follow up at the Lipid Clinic**						
Age first visit, years	55		50 (42–56)		49 (43–54)	0.8
Follow-up, years	55		22 (16–29)		22 (17–27)	0.7
Number of visits, n (%)	55		10 (7–18)		11 (9–15)	0.6
**Biochemical measurements at first visit**						
TC, mmol/L	54		9.30 (7.10–12.10)		8.60 (6.80–11.20)	0.6
TC, highest measured, mmol/L	54		12.50 (10.40–13.00)		12.00 (11.00–13.00)	0.6
LDL-C, mmol/L	48		6.20 (4.60–9.65)		6.93 (4.60–9.00)	>0.9
LDL-C first measure^d^, mmol/L	55		6.20 (4.50–9.07)		6.38 (4.53–8.13)	>0.9
HDL-C, mmol/L	51		1.35 (1.17–1.65)		1.10 (0.94–1.25)	**0.011**
TG, mmol/L	50		1.15 (0.90–1.27)		1.45 (1.04–2.36)	**0.046**
**Biochemical measurements at last visit**						
TC, mmol/L	55		5.00 (4.50–5.60)		3.80 (3.30–4.38)	**<0.001**
LDL-C, mmol/L	55		2.90 (2.40–3.60)		1.90 (1.60–2.48)	**<0.001**
HDL-C, mmol/L	55		1.70 (1.40–1.90)		1.20 (1.03–1.30)	**<0.001**
TG, mmol/L	55		1.10 (0.88–1.25)		1.20 (0.90–1.60)	0.4

Data are presented as n (%) for categorical variables and median (25th-75th percentiles) for continuous variables. Differences between women and men are tested with chi-square test for categorical variables, and Mann-Whitney *U* test for continuous variables. Abbreviations: CHD, coronary heart disease; FH, familial hypercholesterolemia; BMI, body mass index; PCSK9, Proprotein convertase subtilisin/kexin type 9; TC, total cholesterol; LDL-C, low-density lipoprotein cholesterol; HDL-C, high-density lipoprotein cholesterol; TG, triglycerides.

a CHD <55 years in males; <65 years in females.

b Last visit at the Lipid Clinic.

c At least 20 mg Rosuvastatin, 40 mg Atorvastatin or 80 mg Simvastatin.

d First available LDL-C measurement.

### 3.4 LDL-C burden and time-weighted average LDL-C: CHD vs. non-CHD

**Fig. 1:**
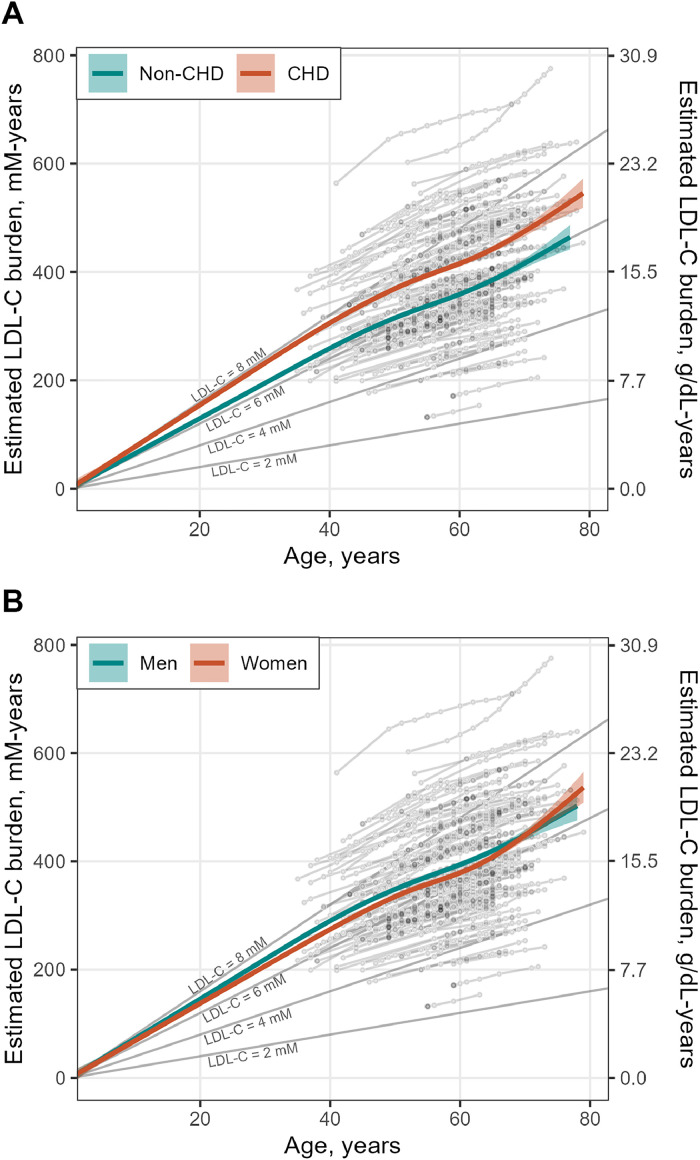
**LDL-C burden in elderly FH patients.** The figure shows estimated LDL-C burden (mM-years and g/dL-years) in A) 55 FH patients with CHD and 57 FH patients without CHD, and in B) 58 female and 54 male FH patients. Individual data points in light grey are connected with lines to highlight the individual trajectories. Green and red lines indicate the average LDL-C burden trends in non-CHD and CHD, respectively (A), and in men and women, respectively (B). Dark grey solid lines represent the LDL-C burden trajectory assuming a time-weighted average plasma concentration of LDL-C of 2 mM, 4 mM, 6 mM and 8 mM.

**Fig. 2:**
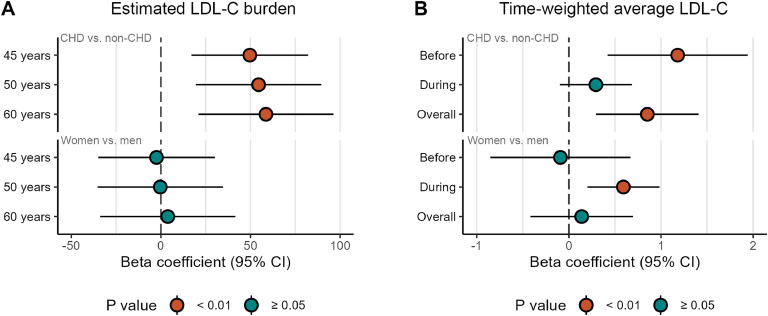
**Differences in LDL-C burden and TWA LDL-C between CHD and non-CHD, and between women and men.** The figure shows differences in estimated LDL-C burden (mM-years) between CHD and non-CHD (upper part of A) and between women and men (lower part of A), at three different age points. Differences in time-weighted average LDL-C (mmol/L) are shown between CHD and non-CHD (upper part of B) and between women and men (lower part of B), before and during treatment at the Lipid Clinic in Oslo, and overall, form birth until last visit. Differences are shown with beta coefficients from linear regression models adjusting for sex when comparing CHD with non-CHD, and adjusting for CHD when comparing sexes. Error bars represent 95 % confidence intervals (95 % CI).

Elderly FH patients with CHD had significantly higher estimated LDL-C burden at all ages tested compared with FH patients without CHD (*p* = 0.004 at 45 and 50 years, and *p* = 0.005 at 60 years), even after adjustment for sex (*p* = 0.003 for all ages), as shown in Fig. 1A, Fig. 2A and Table 4.

**Table 4:** LDL-C burden and time-weighted average LDL-C in elderly FH patients; CHD vs. Non-CHD and Women vs. Men.

	**Overall *n* = 112**	**CHD *n* = 55**	**Non-CHD *n* = 57**	***p***	**Adjusted *p***^1^	**Women *n* = 58**	**Men *n* = 54**	***p***	**Adjusted *p***^2^
**LDL-C burden, mM-years**									
45 years	302 (257–366)	333 (274–409)	281 (244–347)	**0.004**	**0.003**	281 (252–352)	329 (264–386)	0.14	0.9
50 years	329 (282–403)	370 (299–444)	307 (268–380)	**0.004**	**0.003**	309 (276–385)	355 (286–412)	0.2	>0.9
60 years	375 (320–455)	416 (344–486)	358 (307–426)	**0.005**	**0.003**	361 (317–446)	384 (322–474)	0.3	0.8
**TWA LDL-C, mmol/L**									
Overall^3^	5.86 (5.03–6.93)	6.19 (5.40–7.43)	5.54 (4.80–6.61)	**0.008**	**0.003**	5.57 (5.05–6.73)	5.99 (5.02–7.09)	0.4	0.6
Before Lipid Clinic	6.71 (5.70–8.18)	7.20 (5.98–9.20)	6.38 (5.36–7.70)	**0.004**	**0.003**	6.32 (5.56–7.85)	7.30 (5.80–8.75)	0.12	0.8
During follow-up	3.59 (2.90–4.40)	3.67 (3.12–4.44)	3.54 (2.81–4.40)	0.6	0.14	3.93 (3.20–4.55)	3.30 (2.79–4.07)	**0.01**	**0.003**

Data are presented as median (25th-75th percentiles). Differences between CHD and non-CHD, and between sexes, are tested with Mann-Whitney *U* test. Abbreviations: CHD, coronary heart disease; LDL-C, low-density lipoprotein cholesterol; TWA, time-weighted average.

1 Linear regression models with LDL-C burden or TWA LDL-C as outcome and CHD as exposure, adjusted for sex.

2 Linear regression models with LDL-C burden or TWA LDL-C as outcome and sex as exposure, adjusted for CHD.

3 From birth until last visit.

Median overall TWA LDL-C (from birth until last visit) was 6.19 mmol/L in the CHD group, and 5.54 mmol/L in the non-CHD group (*p* = 0.008). While the CHD group had higher TWA LDL-C before their first visit at the Lipid Clinic (*p* = 0.004), there were no difference in TWA LDL-C between the groups during follow-up (*p* = 0.6), see Fig. 2B.

### 3.5 LDL-C burden and time-weighted average LDL-C: women vs. men

There were no statistically significant sex differences in LDL-C burden, by any age, also after adjusting for CHD (Fig. 1B, Fig. 2A and Table 4). However, while the TWA LDL-C was similar between women and men prior to their first visit at the Lipid Clinic, female FH patients had higher TWA LDL-C during follow-up at the Lipid Clinic, with a median of 3.93 mmol/L and 3.30 mmol/L, respectively (unadjusted *p* = 0.01, CHD-adjusted *p* = 0.003), see Fig. 2B.

### 3.6 LDL-C burden and time-weighted average LDL-C at CHD onset

**Fig. 3:**
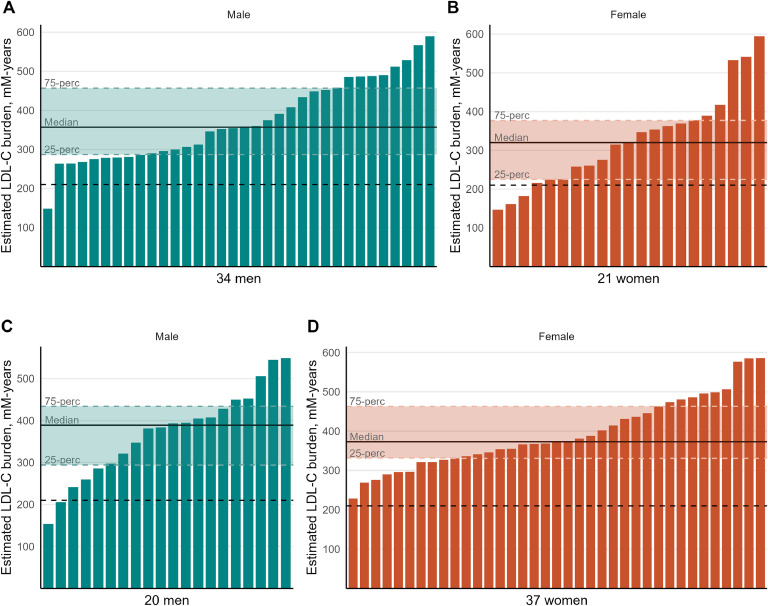
**LDL-C burden at CHD onset in elderly FH patients.** Estimated LDL-C burden (mM-years) at the time of first CHD-event among (A) 34 male and (B) 21 female FH patients with CHD. Estimated LDL-C burden at last visit among (C) 20 male and (D) 37 female FH patients without CHD. Each bar represents an individual's LDL-C burden, and solid black lines represent the median for men and women, with 25–75 percentiles (coloured shading). Dotted black lines represent the theoretical threshold of 210 mM-years for average LDL-C burden at onset of myocardial infarction.

In the CHD group, median estimated LDL-C burden at CHD onset was 352 mM-years (range 147–594 mM-years) and median TWA-LDL-C at CHD onset was 6.94 mmol/L (range 3.66–13.51 mmol/L). See Fig. 3 for the distribution of LDL-C burden at CHD onset in FH patients with CHD, in addition to the LDL-C burden at last visit in FH patients without CHD.

There were no sex differences in LDL-C burden at onset of CHD (*p* = 0.13), even after adjusting for age at CHD onset (*p* = 0.4, Table 5). However, despite no statistically significant differences, women had numerically lower LDL-C burden and age when their first CHD occurred.

**Table 5:** LDL-C burden and TWA LDL-C in FH patients with CHD.

	**Overall *n* = 55**	**Women *n* = 21**	**Men *n* = 34**	***p***	**Adjusted *p***^a^
LDL-C burden at CHD onset, mM-years	352 (277–441)	320 (225–377)	357 (287–457)	0.13	0.4
Age at CHD onset, years	50 (43–56)	45 (40–52)	52 (46–57)	0.13	–
TWA LDL-C at CHD onset^b^, mmol/L	6.94 (6.03–8.92)	6.43 (5.99–9.07)	7.34 (6.18–8.74)	0.7	–

Data are presented as median (25th-75th percentiles). Differences between sexes are tested with Mann-Whitney *U* test. Abbreviations: CHD, coronary heart disease; LDL-C, low-density lipoprotein cholesterol; TWA, time-weighted average.

a Linear regression models with LDL-C burden as outcome and sex as exposure, adjusted for age at CHD onset.

b From birth until CHD onset.

Of the 21 women with CHD, 20 had given birth to a median 2 (range 1–4) children. As all of these women gave birth prior to start of statin treatment, we only took into consideration the LDL-C increase mediated by the pregnancy itself, previously reported to be approximately 30 % [21]. When adding this increase to the overall estimation of LDL-C burden, the LDL-C burden at CHD onset only slightly increased in women, with a median of 324 mM-years (25–75 percentile; 231–379 mM-years). The numerically lower median LDL-C at CHD onset in female compared to male FH patients remained, although not statistically significant (*p* = 0.15).

Of the 55 FH patients with CHD, 4 (7 %) had a LDL-C burden below 210 mM-years (∼8000 mg-years), which is the theoretical average LDL-C burden calculated/suggested by Ference et al., when a person experience myocardial infarction (MI) [5]. All these FH patients had LDL-C burden above the theoretical threshold for LDL-C burden indicating higher risk of MI; 125 mM-years [5]. **Supplementary Table 3** shows characteristics of those with the lowest and highest LDL-C burden at CHD onset. Notably, those with lowest LDL-C burden were female, younger at CHD onset, and started earlier with statin therapy (all *p* < 0.05).

## 4 Discussion

In the present study, we show calculation of estimated LDL-C burden in a group of elderly FH patients, using multiple LDL-C measurements over a period of two decades. Overall, the LDL-C burden was higher in patients with CHD compared with those without CHD, due to higher TWA LDL-C before treatment at the Lipid Clinic. Despite no sex differences in LDL-C burden, female FH patients had higher TWA LDL-C during follow-up and were less intensively treated than males, and, although not statistically significant, women had numerically lower age and LDL-C burden at CHD onset.

Our result of higher estimated LDL-C burden in FH patients with CHD compared to those without CHD is in line with the concept of cumulative effect of LDL-C exposure as a predictor of ASCVD [5]. Korneva et al. have recently reported higher estimated LDL-C burden in middle-aged FH patients with CHD compared to those without CHD, however, the CHD group were significantly older than non-CHD, influencing the results [13]. Also, in a Japanese population of approximately 1000 individuals with clinical FH, Tada et al. showed that the estimated LDL-C burden was higher in those with major adverse cardiovascular events (MACE), but again, individuals with MACE were significantly older than those without [22].

In our study, we compared the LDL-C burden in the CHD and non-CHD groups at similar age points, revealing a differences of 50 mM-years between the groups at the age of 45; a difference that increased to 59 mM-years at the age of 60. Further, our estimated LDL-C burden at these age points made the results comparable to the two aforementioned studies were the CHD-free and MACE-free groups had a mean age close to 45 years, while the groups with CHD and MACE had a mean age of approximately 60 years [13,22]. For CHD-free FH patients at age 45 and FH patients with CHD at age 60, we reported median estimated LDL-C burden of 281 mM-years and 416 mM-years, respectively, relatively comparable to Korneva et al. with LDL-C burden of 261 mM-years and 409 mM-years, and Tada et al. with LDL-C burden of 290 mM-years (11.188 mg-year/dL) and 390 mM-years (15.076 mg-year/dL). Despite the use of different approaches to estimate the cumulative LDL-C exposure in these studies, the present results coinciding with the two previous studies add further indications for the use of estimated LDL-C burden in risk prediction.

Interestingly, we found that the CHD group had higher TWA LDL-C than the non-CHD group before treatment at the Lipid Clinic, but not during follow-up. Although the CHD group received intensive lipid-lowering treatment and reduced their LDL-C to a similar level as the non-CHD group during follow-up, all the years of high LDL-C and hence, higher LDL-C burden prior to treatment, were detrimental. This highlights the importance of early exposure to high LDL-C in contribution to the overall LDL-C burden and cardiovascular risk, and the need for early FH detection and treatment initiation.

Moreover, if previous LDL-C exposure had been considered to guide a more intensive treatment approach, the present FH patients could have gained a markedly lower total LDL-C burden. A recent study showed correlation between estimated LDL-C burden and coronary plaque burden measured with coronary computed tomography angiography (CCTA) in 90 FH patients and 45 controls. They reported that every 75 mM-years increase in LDL-C burden resulted in a two-fold plaque volume increase [14]. Further, Gallo et al. demonstrated that estimated LDL-C burden was a good predictor of cardiovascular events in a French FH cohort followed for 5 years [23]. Although the estimation of cumulative LDL-C exposure in the different studies have varied regarding the calculation approach, age of the FH patients, time of follow-up, and number of available LDL-C measures, it is increasingly evident that such an estimation could aid the assessment of individual susceptibility to CHD. Current guidelines recommend same treatment goal of LDL-*C* < 1.8 mmol/L in adult FH patients regardless of early life LDL-C levels, and even in those attaining treatment goal, high residual risk remains [3,4]. As we observed, most patients with CHD did not reach treatment target. Therefore, estimating LDL-C exposure through simple measures such as LDL-C burden and TWA LDL-C could be valuable in clinical settings, aiding personalized treatment and potentially urge adherence in high-risk individuals [7,23].

We found that women had a higher TWA LDL-C during follow-up at the Lipid Clinic than men. Correspondingly, both we and the FH Studies Collaboration reported a lower goal attainment in female FH patients [24,25]. Further, the present study showed a lower use of potent statin in the women, consistent with previous studies reporting that women are less likely to receive guideline-recommended statin treatment, high intensity statin, and combination therapy when indicated, both in non-FH [26,27] and FH [24,25,28], and in particular at increasing age [29]. Several reasons may cause these sex inequalities. Women are reported to be less adherent to statins due to higher reports of side effects and a poorer belief in the safety and effectiveness [25,26,30,31]. Also, lower CHD rates in women than in men in the general population may contribute to lower awareness of CHD risk in female FH patients, both for healthcare professionals and the women themselves [32]. Sex differences in pharmacological absorption and metabolism may further contribute to the higher average LDL-C in female FH patients under treatment. We have previously shown that girls have higher LDL-C through their childhood and adolescence, as well as higher estimated LDL-C burden at younger ages [33,34], while male FH patients have higher LDL-C levels from the age of 20 years and until female menopause [35]. This is consistent with the present findings of no sex differences in overall LDL-C burden and TWA LDL-C before treatment among elderly FH patients who remained untreated for >40 years. However, as the elderly FH patients we studied were untreated for a long time because of less availability of optimal treatment, a different pattern may be seen in the future. Male patients will be able to adhere to continuous lipid-lowering treatment from treatment start in contrast to women who will have periods of off-statin treatment due to family planning, pregnancy and breastfeeding. Therefore, sex-specific guidelines and increased awareness of cardiovascular risk in women are required to improve prevention and treatment of CHD in female FH patients.

For the first time in elderly FH patients, we estimated LDL-C burden at first CHD-event and compared this to the thresholds suggested by Ference et al. [5]. Only four FH patients had an LDL-C burden below the 210 mM-years threshold at their CHD-event, suggesting a relatively healthy CHD group with few other cardiovascular risk factors besides elevated LDL-C. There were some exceptions, with the minimum estimated LDL-C burden at CHD onset being 147 mM-years, and although not statistically significant, those with lowest LDL-C burden at CHD onset had numerically higher Lp(a) levels. Interestingly, some individuals reached very high LDL-C burdens until first CHD-event occurred, with the highest estimate being 594 mM-years, and despite having lower burden than the CHD group, almost all non-CHD individuals had an overall LDL-C burden above the 210 mM-years threshold at their last visit. These findings likely reflect that CHD is a multifactorial disease, influenced by several other risk factors besides LDL-C [12]. We previously reported larger HDL particles and different expression of lipid and immune related genes in seemingly CHD resistant elderly FH patients [36,37]. Combined with the present findings of lower TWA LDL-C during childhood and early adulthood in the non-CHD group, this supports Domanski and colleagues’ report of greater cardiovascular risk with high cumulative LDL-C exposure early in life compared with later in life [10], potentially inducing changes in immune responses and lipid metabolism that persist even after LDL-C reduction. Nevertheless, unravelling cardioprotective mechanisms in individuals who tolerate high LDL-C burden is essential in future research, and a potential LDL-C threshold to guide treatment should be combined with other risk scores.

Despite no statistically significant sex differences in LDL-C burden at CHD onset, there were trends towards lower burden, TWA LDL-C and age at CHD onset among women. These trends persisted after accounting for the normal LDL-C increase during pregnancy and were not explained by other cardiovascular risk factors. Sex differences in vascular physiology and plaque characteristics, and higher aspirin-resistance in women, are previously shown [[38], [39], [40]], and further investigation into potential sex differences in LDL-C burden thresholds for increased CHD risk is needed.

### 4.1 Strengths and limitations

The main strength of this study is the high numbers of LDL-C measurements over the course of two decades of follow-up at the Lipid Clinic, providing a robust estimation of LDL-C burden. Generally, repeated measurements of risk factors have previously been shown to improve risk prediction compared to a single measurement of the same risk factors, which is used in standard risk scores [41]. With all the available measurements, we could follow the different LDL-C trajectories in FH patients during late adulthood, and for the first time provide an estimation of LDL-C burden at CHD onset in FH patients using real-world data. Further, calculation of TWA LDL-C allowed for an even more comprehensible approach to the lifelong LDL-C exposure, enabling the findings of sex differences in LDL-C during follow-up for FH patients. Of notice, 97 % of all participants had genetically confirmed FH mutation.

A limitation in our study is the use of later-life LDL-C measurements to predict the earlier-life LDL-C levels and burden, not accounting for lipid-affecting factors such as diet or puberty, and general changes in LDL-C during early life. However, our estimates are based on a median of nine measurements, higher than in comparable studies [13,14,22], and provide promising results for using available LDL-C levels from electronic medical records in CHD risk prediction in FH patients. Further, due to statin use at first LDL-C measure without information on type or dose, we estimated untreated LDL-C in approximately half of the FH patients based on the assumption of 30 % reduction with statins. This approach did not account for variability in drug prescription, drug response or adherence. However, the method is previously used by others [11,17,18], and across all the comparing groups, similar proportions of participants required calculation of untreated LDL-C, indicating minimal impact on the results. Using a different approach to calculate untreated LDL-C in sensitivity analysis produced similar results [19], however, the second approach slightly elevated the absolute level of LDL-C burden in all groups, hence, the numerical LDL-C burden at CHD onset should be interpreted with caution. Further, the lack of statistically significant sex differences in LDL-C burden at CHD onset might be due to relatively few participants. Overall, this observational single-center study consisted of a relatively small sample of Norwegian FH patients, hence, larger studies including other FH populations and non-FH populations are needed to validate and generalize our findings.

In conclusion, our findings of higher overall LDL-C burden together with higher TWA LDL-C *before* treatment among those with CHD, and higher TWA LDL-C *during* treatment among women, underscore the importance of both early initiation and more intensive lipid-lowering treatment in FH patients in general, and to treat women equally well as men, to minimize the total LDL-C exposure and thus reduce the lifetime risk of CHD. Further, we emphasize the usefulness of estimated LDL-C burden and the perhaps more understandable TWA LDL-C in CHD prevention and communication with the patients. There is a need for more individualized treatment target in those with already acquired high exposure to LDL-C, as well as increased focus on female FH patients.

## Funding

This work was supported by the National Health Association, Norway; University of Oslo, Norway; the Norwegian National Advisory Unit on FH, Oslo University Hospital, Norway; and the Throne-Holst Foundation for Nutrition Research, Oslo, Norway.

## CRediT authorship contribution statement

**Torunn Melnes:** Writing – review & editing, Writing – original draft, Visualization, Resources, Methodology, Formal analysis, Data curation, Conceptualization. **Martin P. Bogsrud:** Writing – review & editing, Writing – original draft, Supervision, Methodology, Investigation, Formal analysis, Data curation, Conceptualization. **Jacob J. Christensen:** Writing – review & editing, Methodology, Formal analysis. **Amanda Rundblad:** Writing – review & editing, Methodology, Formal analysis. **Kjetil Retterstøl:** Writing – review & editing, Investigation. **Ingunn Narverud:** Writing – review & editing, Data curation. **Pål Aukrust:** Writing – review & editing, Investigation. **Bente Halvorsen:** Writing – review & editing, Investigation. **Stine M. Ulven:** Writing – review & editing, Investigation. **Kirsten B. Holven:** Writing – review & editing, Writing – original draft, Supervision, Resources, Methodology, Funding acquisition, Conceptualization.

## Declaration of competing interest

Kirsten B Holven, reports a relationship with Sanofi, Norway that includes: speaking and lecture fees. Martin P. Bogsrud reports a relationship with Sanofi, Norway that includes: consulting or advisory. Kjetil Retterstøl reports a relationship with Sanofi, Norway that includes: consulting or advisory. Martin P. Bogsrud reports a relationship with Amgen, Norway that includes: consulting or advisory. Kjetil Retterstøl reports a relationship with Amgen, Norway that includes: consulting or advisory. Jacob J Christensen reports a relationship with Amgen, Norway that includes: consulting or advisory. Jacob J Christensen reports a relationship with Mills DA, Norway that includes: consulting or advisory. Kjetil Retterstøl reports a relationship with Mills DA, Norway that includes: consulting or advisory. Amanda Rundblad reports a relationship with Rimfrost AS, Norway that includes: consulting or advisory. Kjetil Retterstøl reports a relationship with Akcea, Norway that includes: consulting or advisory. Kjetil Retterstøl reports a relationship with Sunnovion, Norway that includes: consulting or advisory. The other authors declare that they have no known competing financial interests or personal relationships that could have appeared to influence the work reported in this paper.

## Appendix Supplementary materials

Image, application 1
